# Supplementary material for: The genome sequence of a braconid wasp, Aleiodes leptofemur van Achterberg & Shaw, 2016
Source: Wellcome Open Res. 2024 Aug 12;9:460. [Version 1] doi: 10.12688/wellcomeopenres.22769.1 (PMC11416704; doi:10.12688/wellcomeopenres.22769.1)
Supplement: Peer review history

# Reviewer report: Reviewer response for version 1

**DOI:** 10.21956/wellcomeopenres.25073.r96679

**Published:** 2024-09-21

**Authors:** Julien Varaldi

**Affiliations:** 1 Universite Claude Bernard Lyon 1, Villeurbanne, France

This manuscript presents the complete chromosome-level assembly of *Aleiodes leptofemur* (braconid wasp; Arthropoda; Insecta; Hymenoptera; Braconidae). This is a parasitoid wasp that attacks Lepidoptera caterpillars. The manuscript is clear and concise and the analysis is sound. The final assembly is of high quality thanks to the use of short and long reads and HiC data obtained from the DNA of a single (haploid) male.

**I have only very minor comments:**

- I think the peculiar way of life (parasitoid) of this species is not clearly stated in the introduction. A few additional details on its biology could also be useful to the reader. For instance is it an endo- or an ecto-parasitoid? a solitary or gregarious parasitoid? It would also have been nice to know why the authors focused on this particular species.
- Please provide a reference for this assertion, if available. “The winter is spent as a larva in the host which is often manipulated to climb to an exposed position before being killed.”
- There is a bit of inconsistency in the coverage values provided in the result section.
- It is mentioned that 14.67Gb of PacBio reads have been produced, giving a 59-fold coverage: The genome is thus expected to be 14.67/59 = 0.2486441 Gb.
- Next sentence, similar reasoning is given for Hi-C reads but this leads to a slightly different value: 116.45Gb/429=0.2714452Gb

Are sufficient details of methods and materials provided to allow replication by others?

Yes

Is the rationale for creating the dataset(s) clearly described?

Partly

Are the datasets clearly presented in a useable and accessible format?

Yes

Are the protocols appropriate and is the work technically sound?

Yes

Reviewer Expertise:

evolutionary biology, genomics, horizontal gene transfer

I confirm that I have read this submission and believe that I have an appropriate level of expertise to confirm that it is of an acceptable scientific standard.

# Reviewer report: Reviewer response for version 1

**DOI:** 10.21956/wellcomeopenres.25073.r96678

**Published:** 2024-09-18

**Authors:** Andrew D Austin

**Affiliations:** 1 The University of Adelaide, Adelaide, South Australia, Australia

This is a very competent piece of research which presents a fully annotated genome of a braconid wasp. As such it will be highly useful for a plethora of future studies on the biology and evolution of the group.  The methods are well articulated and easy to follow, and the data clearly presented.

 I have but two suggestions to improve the paper:

1. First a little more detail about the characters helpful for identifying the subfamily and genus would assist anyone not familiar with the group.
2. Second, a statement at the end of the introduction regarding the rationale for choosing this particular genus/species AND the significance of this genome for guiding future studies would be useful. My understanding is that there are very few fully annotated genomes for braconid wasps, and so this study is of particular importance.

Are sufficient details of methods and materials provided to allow replication by others?

Yes

Is the rationale for creating the dataset(s) clearly described?

Partly

Are the datasets clearly presented in a useable and accessible format?

Yes

Are the protocols appropriate and is the work technically sound?

Yes

Reviewer Expertise:

molecular phylogenetics, evolution and systematics of parasitic wasps

I confirm that I have read this submission and believe that I have an appropriate level of expertise to confirm that it is of an acceptable scientific standard.
